# The burden of trauma in the life of a refugee

**DOI:** 10.3389/fpubh.2024.1298544

**Published:** 2024-07-17

**Authors:** Samina Salim

**Affiliations:** Department of Pharmacological and Pharmaceutical Sciences, College of Pharmacy, University of Houston, Houston, TX, United States

**Keywords:** trauma, refugee, Gaza, Ukraine, Palestine

## Introduction

The word “refuge” carries a kind and comforting connotation, but the word “refugee” is immediately equated with a stigma. This is unfortunate considering the current unstable geopolitical environment of the world, where global conflicts are resulting in mass displacements of communities and increasing the number of refugees to an unprecedented proportion (1–3). Once displaced, affected communities embark on a complex new terrain of migration and resettlement in the host countries, often facing economic disparities, health and food insecurity, racism, stigma, and absence of or poor access to healthcare (3, 4). For example, studies show a close association between war-related trauma and post-migration stress of refugees, with increased rates of both physical (metabolic ailments, and cardiovascular disease) and mental health problems, such as post-traumatic stress disorder (PTSD), depression, and anxiety (5–14).

## Trauma, displacement, and resettlement

We and others have shown that moving away from the war zone does not erase traumatic memories or restore the mental health of refugees. For example, our study of Houston-based adult Syrian refugees who had left their home country years before resettling in Houston, continue to exhibit PTSD (15). This is worse among children, 52% of whom in our un-published study sample exhibited symptoms of PTSD. We also found that Syrian refugee women reported significantly higher stress and distress than men (15). This is consistent with the theory that women often repress their mental health needs due to family responsibilities, social stigma, and/or cultural pressures. In our study, displacement from home country and social strain were the highest source of stress among women, as indicated using the refugee post-migration stress scale (15). This and other studies show the consequences of refugee trauma are long-lasting (16–20).

The process, and the circumstances of displacement may vary but the unifying theme among all refugees is the trauma of uprootment and the stigmatization following resettlement. Host communities often perceive refugees as a burden on the economy. This mindset combined with the otherization factor often contributes to isolation of these vulnerable groups eliciting negative mental health outcomes. The culmination of the multitude of stressors encompassing physical, psychosocial, and socio-economic factors, that occur during displacement and post-resettlement in host environments, often results in permanent psychological damage to the mental health of refugees. In addition, the trauma often continues to exist, although vicariously, and the burden of trauma may transcend to future generations. Children of the refugees who witnessed trauma and those who hear about traumatic experiences from their parents often continue to carry this burden as they face the new challenges of integrating into the new school system, combined with the pressure of assimilating into a new culture. Not surprisingly, mental health problems, including depression, PTSD, and traumatic grief are commonly reported in refugee children born in the host country (21–26). These conditions are usually accompanied with academic problems, and often with behaviors increasing health-risk (e.g., smoking, eating disorders, substance use) and accompanied by long-term health problems (e.g., diabetes and heart disease).

## The geopolitical environment

On an introspective note, the refugee crisis on some level, is a result of our complicity in global wars which western governments fund at tax payers expense for global dominance as well as for political, and economic interests (27–29). Our disengagement from civic and political processes and our inability to hold our governments accountable often results in use of citizen's taxes to forge sale of lucrative arms and weapons to countries that are openly committing acts of aggression and incurring a great cost in human lives, while causing mass displacement of men, women and children (3, 30, 31). According to the United Nations High Commission of Refugees (UNHCR), we are experiencing the highest levels of displacement on record with more than 114 million individuals remain forcibly displaced worldwide as a result of persecution, war, conflict, violence or human rights violations (3). There exists great promise of promoting peace through science, therefore, I suggest using science as a soft power to build bridges. This can be achieved by providing scientific advice and evidence to inform and support healthcare needs of those that lie at the receiving end of the political fallouts of global crisis such as the ongoing wars in Ukraine, Gaza, and Rafah.

## War and conflict

For a long time, I hoped there would be no crisis more devastating than the Syrian refugee crisis, but the Russian invasion of Ukraine and the Israel-Hamas war has proved otherwise. These two major world events will most-likely be the most consequential events in modern history. The Russian invasion has led to one of the largest refugee crises of our times, with one in four Ukrainian citizens displaced from their homes contributing to an estimated 4 million displaced population. This is especially concerning for the older adult population, people with chronic diseases, and women and children, who are the most vulnerable among the displaced communities. According to the United Nations Children's Fund (UNICEF), an estimated 5 million Ukrainian children are displaced from their homes (32). According to most accounts this number is more than half of Ukraine's children. With the men at war, women make up 80% of the adult population of refugees (18+), including half of the women aged 25–44 years (33). Although humans are inherently resilient, the mental toll of trauma from war, conflict, displacement and resettlement is unavoidable, which will persist for a long time in some and last forever in others (34). Research conducted in Ukrainian refugees resettled in Poland have suggested significant anxiety, depression, and sleep disturbances (35–38). Another matter of grave concern that has emerged from this war is the issue of racism evident from how the world has reacted to this crisis *vis a vis* the global refugee crises which brings to the forefront the long standing bias associated with skin color (10, 39–49). This became evident during the early phases of the Ukrainian refugee crisis when some journalists, political commentators, and some of the public implied that since Ukrainians are Europeans i.e., White, they deserve more dignity and respect than people of color (10, 39–49), or referring to the situation as “it is not a third-world nation.”. Or other racist phrases such as “we are not in the Middle East or Africa” or that “Ukrainian refugees are civilized,” from the “middle-class,” like “any European family,” with “blue eyes and blond hair.” These statements not only trivialized the suffering of others but brought to light the deep sense of racism still prevelant in modern societies (10, 39–49). It is reported that refugees of color from Ukraine now carry a double trauma: the burden of war and that of racism. This type of racial trauma emcompassing prejudice, and humiliation combined with various types of racial discrimination (38, 41–48) has been associated with PTSD, depression, anxiety, increased suicidal ideation and suicide attempts, hypertension, diabetes, heart disease, etc. (37, 49). With the current stalemate in the Ukraine-Russian situation and with no resolution in sight, the plight of Ukrainian refugees is expected to deteriorate.

The situation in Gaza Strip, Palestine, home to 2.4 million residents, is worse, as a grave humanitarian crisis is unoflding, with civilians particularly women and children, bearing the brunt of this crisis (50, 51). According to recent estimates, on an average, 420 children are killed or injured daily, with a child dead every 15 min (52). According to Council of Foreign Relations (cfr.org), 75% of Gaza's population is currently displaced, the majority of whom are crammed in Rafah, considered the last designated safe zone for those seeking refuge, which despite international condemnation and calls for restraint, is also under attack by Israeli forces.

Egypt, which shares the Rafah border crossing with Gaza already hosts an estimated 390,000 refugees and asylum seekers. Large numbers of Palestinians have been refugees since the Arab-Israel wars of 1948, and 1967. They have been displaced from their native lands and resettled in refugee camps in the West Bank and Gaza Strip, which are no longer deemed temporary (50). Making things worse, destruction of healthcare facilities, water and electricity shortage, food insecurity and lack of access to life saving medications severely impede medical emergencies and healthcare (51–60). In general, the socio-economic impact of war on displaced communities during times of war is huge but the psychological toll it can take on internally displaced communities temporarily shelterd in refugee camps can be disastrous. Reports of post-traumatic stress, anxiety, depression, behavioral issues, emotional disorders, and desensitization to violence among Gazans are emerging (51–60). It will be interesting to see how the world reacts to the Gaza refugee crisis *vis a vis* the Ukrainian refugee crisis once the Gazans face war fatigue and begin fleeing for safety.

Another significant matter worth serious examination is the issue of transgenerational trauma, which Jewish and Palestinian communities have both suffered from. The aftermath of the Holocaust and centuries of displacement and persecution of Jewish communities has reportedly resulted in transgenerational trauma in this group (61–76). Extensive studies conducted with the offspring of Holocaust survivors who endured traumatic experiences in labor camps and residential ghettos, faced family separation, and starvation, among other cruelties under Nazi occupation in Europe during World War II, emphasize presence of transgenerational trauma among Jewish communities worldwide (61–76). And, more than 76 years of forced displacement, dispossession, oppression, occupation and poor socio-economic conditions (51–60, 77–79) of Palestinians, also has resulted in a complex form of transgenerational trauma, characterized by a deep sense of injustice, anger and unrest. This is expected to worsen and be further complicated by the war in Gaza.

## Second hand trauma

Among the many disastrous consequences emerging from the recent Israel-Hamas, and the Russia-Ukraine war is the unique nature of the collective mental trauma felt across societies globally. In this era of high-speed digital dissemination of information in real time, the Israeli military aggression in Gaza, the rising death toll and the dibilatated state of humanity, are watched and read across all seven continents 24-7, leaving societies feeling overwhelmed with grief, anger, frustration and fear. This is particularly problematic for vulnerable inidviduals who might relive their own traumatic memories from witnessing images of Israeli aggression, death and destruction in Gaza on their television screens and on their phones. This may be particularly alarming for the mental health of people who have been previously exposed to war, displacement, oppression, and armed conflict (77–79) as their emotional sensitivity to such events may be exacerbated from current genocidal situation in Gaza. Thus, the secondary trauma of this nature may be critically consequential for the state of global mental health.

## Promotion of peace and diplomacy through science

Refugee health is a complex matter, the one which is critically dependent upon psychosocial, and economic determinants deserving a multipronged approach to achieve positive individual health outcomes and broader societal outcomes. Small changes can have a big impact. For example, the “otherization” mindset may be challenged by doing something as simple as revising basic definitions. Relevant to this, the United Nations High Commissioner for Refugees (UNHCR) describe refugees as “people who have fled war, violence, conflict or persecution and have crossed an international border to find safety in another country.” While the definition may accurately describe the status of the displaced group, it does not emphasize what appears to be the most obvious and perhaps the most critical aspect of a refugee life i.e., refugees are civilians without the means of self-protection and/or defense who are forced to abandon their homes and in some cases unwillingly leave their loved ones behind. Revising the definition as “people who have fled war, violence, conflict, or persecution and have unwillingly left their homes, relatives, and properties to find safety in another country” is a more inclusive and accurate description of a refugee situation. This amendment might mitigate otherization and stigmatization often faced by refugees who unfortunately are simply “collateral damage” of a political fallout; who in a matter of few hours find themselves living in a situation of high peril, sleeping in communal housing on benches far away from the comfort of their warm bed and the safety of their homes. Refugee-focused outreach programs rooted in local communities led by local community leaders may offer sustainable and impactful solutions. Culturally informed and language-tailored mental health awareness programs, combining in-person with e-health dissemination channels, have the potential to increase awareness, improve mental health screening uptake while reducing morbidity and mortality and improving quality of life.

There remain opportunities where scientific exchange can promote interaction and understanding across borders. For example, fostering peace between Israelis and Palestinians is a much needed, although complex, yet a necessary goal. This conflict which has raged since the founding of the modern Jewish state in 1948 has caused extensive trauma for both Jewish and Palestinian communities. Serious international efforts to promote justice, interaction, collaboration and peace-building through scientific and medical initiatives is needed. In the past, some initiatives were put into place which at present are stalled or ended (80–84).

Finally, science is not only a safe space for building international coalitions and for forging innovative collaborations, but science can also be leveraged as an excellent mechanism for creating good will on which diplomatic relations can be built to promote a long-lasting peace, especially teams of “scientists without borders” can play a pivotal role in serving global health interests. While the ideological differences determining global politics may be large, academic connections can go a long way in advancing science and promoting peace.

## Author contributions

SS: Conceptualization, Data curation, Funding acquisition, Investigation, Methodology, Project administration, Resources, Supervision, Writing – original draft, Writing – review & editing.
